# The association between the cardiometabolic index and hyperuricemia: 2011–2016 NHANES

**DOI:** 10.3389/fendo.2025.1545968

**Published:** 2025-05-13

**Authors:** Yishan Zhou, Yihong Zou, Yuyu Cao, Yang Gao, Ying Pi, Xiaona Tang, Yang Liu, Yanghong Zhong

**Affiliations:** Seventh Clinical Medical College, Guangzhou University of Chinese Medicine, Shenzhen, China

**Keywords:** NHANES, central obesity, cardiometabolic index, hyperuricemia, serum uric acid

## Abstract

**Background:**

Central obesity is associated with hyperuricemia. However, the association between the cardiometabolic index (CMI), which incorporates abdominal obesity and lipid metabolism parameters to assess central obesity, and hyperuricemia, is unclear. This study aimed to explore the association between CMI and hyperuricemia.

**Methods:**

We enrolled 5,338 study participants from the 2011–2016 National Health and Nutrition Examination Survey. The participants were divided into three groups based on tertiles of CMI. We performed linear regression and employed weighted logistic regression models, subgroup analyses, and restricted cubic spline (RCS) regression to investigate the association between CMI and hyperuricemia.

**Results:**

In this study, 20.4% of the participants were diagnosed with hyperuricemia. A higher CMI correlated consistently with increased serum uric acid levels (β: 0.51; 95% confidence interval (CI): 0.38–0.64) and hyperuricemia (odds ratio (OR): 2.62; 95% CI: 1.91–3.61). In the fully corrected model, for each unit increase in CMI, the incidence of hyperuricemia increased by 54% and serum uric acid levels increased by 0.28 mg/dL. Subgroup analyses showed that the association of CMI with hyperuricemia was stably present in all subgroups. Interaction effects were observed for sex and body mass index subgroup (*p* for interaction: < 0.05). RCS regression highlighted a significant positive nonlinear association (*p* < 0.001).

**Conclusion:**

Our results indicated a significant positive association between CMI levels and hyperuricemia risk among US adults.

## 1 Introduction

Uric acid is the final metabolite of purine metabolism, and increases in its production or excretion dysfunction will lead to elevated serum uric acid (SUA) levels and even develop into hyperuricemia (1). With the improvement of modern living standards and changes in dietary patterns, the prevalence of hyperuricemia is increasing year by year (2). Most hyperuricemia has no symptoms in the early stage, and if not controlled in time, will lead to gout, kidney stones, uric acid nephropathy, and other diseases (3, 4). However, there is no clear index that can predict hyperuricemia; therefore, finding an index that can predict SUA levels is essential to reduce the risk of hyperuricemia and improve the quality of life of patients.

Risk factors for hyperuricemia include insulin resistance, obesity, hyperlipidemia, and atherosclerosis, among which obesity is considered a particularly important factor (5–7). Previous studies showed that obesity and abnormal lipid metabolism correlated with SUA levels, which could be significantly reduced when obesity or dyslipidemia were treated (8, 9). This evidence suggests that obesity and the lipid metabolic state have an impact on SUA levels. Therefore, recommendations for the management of hyperuricemia usually emphasize the role of weight loss (8). Obesity can be subdivided into central obesity and peripheral obesity. Central obesity is characterized by increased abdominal and central fat, which is associated with a variety of metabolic disorders, and is more closely related to uric acid than peripheral obesity (10, 11). A study conducted in Korea investigated the relationship between abdominal fat and SUA in patients with diabetes, which showed that the abdominal fat area correlated positively with SUA levels, representing a significant independent predictor of hyperuricemia; however, there was no significant correlation between the subcutaneous fat area and SUA levels (12).

In 2015, the cardiometabolic index (CMI) (13) was proposed by Ichiro Wakabayashi as a novel index to assess central obesity, which has been shown to be associated with a variety of metabolic disorders, combining abdominal obesity and lipid metabolism parameters, and predicting the risk of diabetes mellitus, coronary artery disease, and metabolic syndrome brought on by obesity and dyslipidemia (14, 15). As demonstrated by a cross-sectional study in China, the CMI predicts hyperuricemia with normal body mass index (BMI) and is more accurate compared with the visceral adiposity index, body adiposity index, and lipid accumulation product (16). Another study showed the CMI might be a monitoring indicator of hyperuricemia in overweight people (17). In addition, The CMI can predict hyperuricemia among middle-aged and elderly populations (18). However, existing studies on CMI and hyperuricemia do have limitations in population representation and extrapolation of conclusions. Indeed, evidence of the relationship between the CMI and SUA levels remains weak in the US general population. Therefore, we designed this cross-sectional study to assess the correlation between CMI and SUA levels among adults in the USA, which might represent a predictor of SUA levels and provide a theoretical basis for the management of patients with hyperuricemia.

## 2 Materials and methods

### 2.1 Study population

Our data were obtained from the National Health and Nutrition Examination Survey (NHANES), a comprehensive cross sectional study administered by the US National Center for Health Statistics (NCHS). It assesses the health and nutritional status of the US population by collecting data through questionnaires, interviews, physical tests, and laboratory examinations. For more information on the data, visit (https://www.cdc.gov/nchs/nhanes/index.htm). The methods and materials used in the survey were approved by the NCHS Ethics Committee, and all information collected in the survey is kept strictly confidential. Privacy is protected by public laws.

**Figure 1:**
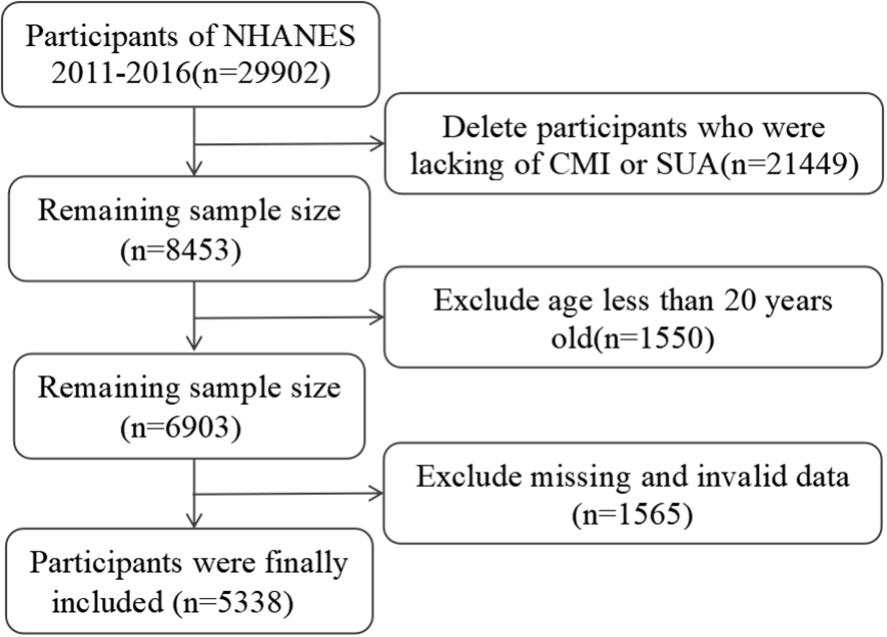
Flowchart of the selection of study participants.

A total of 29,902 participants were included from 2011 to 2016, and we excluded 21,449 patients with missing CMI or SUA data, in addition to excluding 1,550 participants younger than 20 years of age, and finally deleting 1,382 participants with missing covariates. Ultimately, 5,338 participants were included in the study (**Figure 1**).

### 2.2 The cardiometabolic index

The CMI consists of lipid metabolism and body measurements indicators, calculated by the following formula: (13)


$$\text{CMI}=\frac{\text{TG}(\text{mmol}/\text{L})}{\text{HDL}‐\text{C}(\text{mmol}/\text{L})}\times \frac{\text{waist circumference}(\text{cm})}{\text{height}(\text{cm})}$$

where TG is triglycerides and HDL-C is high-density lipoprotein-cholesterol. The participants were divided into three groups according to their CMI tertiles (T): T1 (CMI < 0.3368), T2 (CMI = 0.3368 to 0.6875), and T3 (CMI > 0.6875).

### 2.3 Serum uric acid level and hyperuricemia

As the dependent variable in this study, the SUA level was measured using a Beckman Coulter UniCel^®^ DxC800 instrument (Beckman Coulter Indianapolis, IN, USA). Hyperuricemia was defined as an SUA level ≥ 7.0 mg/dL in males and ≥ 6.0 mg/dL in females (19).

### 2.4 Covariates

Covariates included age, sex, race, education level, marital status, income-to-poverty ratio (PIR), body mass index (BMI), smoking status, alcohol drinking status, total cholesterol (TC), low-density lipoprotein cholesterol (LDL-C), blood urea nitrogen (BUN), chronic kidney disease (CKD) history, and cardiovascular disease (CVD) history.

There were three categories for educational attainment: below, equal to, and above high school. Marital status was categorized as ‘living with a partner or married’ and ‘single, divorced, or widowed.’ The BMI was calculated as weight (kg) divided by squared height (m) and categorized as < 25.0 and ≥ 25.0 kg/m^2^ (20). The response “at least 100 cigarettes smoked in a lifetime” was used to establish smoking status (21). Whether or not they drank at least 12 drinks annually was considered to indicate their drinking status (22). Participants with CKD were defined as having a glomerular filtration rate < 60mL/min/1.73m^2^, an albumin creatinine ratio ≥ 30 mg/g, based on the laboratory tests, or they had been previously diagnosed with CKD based on the questionnaire (23). Participants were considered to have CVD if they had suffered from any of congestive heart failure, coronary heart disease, angina pectoris, heart attack, or stroke, in accordance with the Medical Conditions section of the questionnaire (24).

### 2.5 Statistical analysis

According to the NHANES recommended sample weight on Fasting Subsample 2 Year MEC Weight (WTSAF2YR) records, sample weights of individuals were determined by WTSAF2YR/3. Continuous variables are presented as mean ± SD or median (interquartile range), depending on the distribution. Categorical variables were expressed using numbers (percentage, %). Weighted linear regression and logistic regression modeling were used to investigate the relationship between the CMI and different outcomes (SUA level and hyperuricemia). Furthermore, weighted restricted cubic spline regression curves were created and subgroup analyses were also performed. We conducted subgroup and interaction analyses with participants stratified by sex (male/female), age (< 60/≥ 60 years old), BMI (< 25.00/≥ 25.00), drinking status, smoking status, CKD(no CKD and CKD) and CVD (no CVD and CVD).

In Model 1, no covariates were adjusted for. In Model 2, adjustments were made for age, sex, and race. In Model 3, adjustments were made for age, sex, race/ethnicity, smoking and drinking status, BMI, TC, LDL-C, BUN, PIR, education level, marital status, CKD, and CVD history. All statistical analyzes were performed using SPSS (version 25.0) and Free Statistics (version 2.1.1). A two-sided *p* < 0.05 was considered statistically significant.

## 3 Results

### 3.1 Baseline characteristics

We enrolled 5,338 participants, of whom 48.81% were male, and their median age range was 47 (33–61) years old. **Table 1** shows a progressive increase in SUA levels and with an increasing CMI, which might be accompanied by CVD. The prevalence of CKD and CVD among participants was 2.55% and 8.60%, respectively. The highest CMI tertiles had higher BMI, TC, LDL-C, BUN, higher cigarette use, and a lower PIR compared with the lowest CMI tertiles. Moreover, there were significant differences between the three tertiles in terms of race, education level, and marital status, but no statistically significant differences in drinking status and CKD.

**Table 1:** Baseline characteristics of the participants according to the tertiles of the cardiometabolic index (weighted).

CMI	Total N =183,543,104 n = 5338	T1 N =62,040,212 n = 1781	T2 N =60,444,222 n = 1778	T3 N =61,058,671 n = 1779	p -value
SUA(mg/dL)	5.40 (4.50, 6.30)	4.80 (4.10, 5.70)	5.40 (4.50, 6.21)	6.00 (5.10, 6.90)	< 0.001
Hyperuricemia, n (%)					< 0.001
No	4,226 (79.60)	1,602 (90.15)	1,423 (81.97)	1,201 (66.53)	
Yes	1,112 (20.40)	179 (9.85)	355 (18.03)	578 (33.47)	
Age	47.00 (33.00, 61.00)	42.81 (29.00, 60.00)	48.00 (34.00, 60.00)	49.69 (37.00, 62.00)	< 0.001
Gender, n (%)					< 0.001
Male	2,632 (48.81)	724 (39.67)	887 (49.02)	1,021 (57.89)	
Female	2,706 (51.19)	1,057 (60.33)	891 (50.98)	758 (42.11)	
BMI(kg/m^2^)	28.00 (24.30, 32.70)	24.40 (22.00, 27.90)	28.10 (25.00, 32.40)	31.70 (28.00, 36.10)	< 0.001
Race/ethnicity, n (%)					< 0.001
Mexican American	713 (7.98)	159 (5.73)	251 (8.28)	303 (9.98)	
Other Hispanic	573 (5.74)	130 (4.32)	210 (6.26)	233 (6.67)	
Non-Hispanic white	2,237 (68.17)	707 (66.37)	719 (67.90)	811 (70.26)	
Non-Hispanic black	1,067 (10.71)	490 (15.10)	362 (10.66)	215 (6.30)	
Non-Hispanic Asian	599 (4.68)	244 (5.78)	193 (4.59)	162 (3.66)	
Other race	149 (2.72)	51 (2.71)	43 (2.31)	55 (3.13)	
Income to poverty ratio	2.92 (1.42, 4.94)	3.20 (1.53, 5.00)	2.82 (1.42, 4.90)	2.65 (1.31, 4.61)	< 0.001
Education level, n (%)					<0.001
Less than high school	1,127 (15.59)	296 (12.20)	398 (16.89)	433 (17.75)	
high school	1,128 (19.36)	333 (17.23)	386 (19.41)	409 (21.49)	
More than high school	3,083 (65.05)	1,152 (70.57)	994 (63.70)	937 (60.77)	
Marital status, n (%)					<0.001
Married/living with partner	3,238 (64.62)	1,007 (63.36)	1,086 (63.86)	1,145 (66.67)	
Single/divorced/widowed	1,106 (17.03)	323 (13.46)	388 (18.50)	395 (19.19)	
Never maeeied	994 (18.35)	451 (23.18)	304 (17.64)	239 (14.13)	
TC(mmol/L)	4.86 (4.19, 5.56)	4.65 (4.03, 5.33)	4.89 (4.24, 5.56)	5.07 (4.37, 5.82)	< 0.001
LDL-C(mmol/L)	2.87 (2.28, 3.52)	2.61 (2.10, 3.16)	2.97 (2.43, 3.60)	3.03 (2.41, 3.70)	< 0.001
BUN(mg/dL)	13.00 (10.00, 16.00)	12.00 (10.00, 16.00)	13.00 (10.00, 16.00)	13.00 (11.00, 16.00)	0.003
Chronic kidney disease, n (%)					0.120
Yes	185 (2.55)	44 (2.17)	59 (2.29)	82 (3.20)	
No	5,153 (97.45)	1,737 (97.83)	1,719 (97.71)	1,697 (96.80)	
Cardiovascular disease, n (%)					< 0.001
Yes	546 (8.60)	130 (6.07)	178 (7.83)	238 (11.94)	
No	4,792 (91.40)	1,651 (93.93)	1,600 (92.17)	1,541 (88.06)	
Smoking ≥100 cigarettes in life, n (%)					<0.001
Yes	2,337 (44.12)	666 (36.81)	783 (44.87)	888 (50.81)	
No	3,001 (55.88)	1,115 (63.19)	995 (55.13)	891 (49.19)	
Alcohol drinking status, n (%)					0.620
Yes	3,877 (78.13)	1,311 (78.93)	1,280 (77.64)	1,286 (77.81)	
No	1,461 (21.87)	470 (21.07)	498 (22.36)	493 (22.19)	

CMI, Cardiometabolic index; SUA, serum uric acid; BMI, body mass index; TC, total cholesterol; LDL-C, low-density lipoprotein cholesterol; BUN, blood urea nitrogen; T, tertile; n, unweighted; N, weighted.

T1, 0.04≤ CMI< 0.3368; T2, 0.3368≤ CMI< 0.6875; T3, 0.6875≤ CMI≤ 12.04.

### 3.2 Association between the CMI and SUA level/hyperuricemia

**Figure 2:**
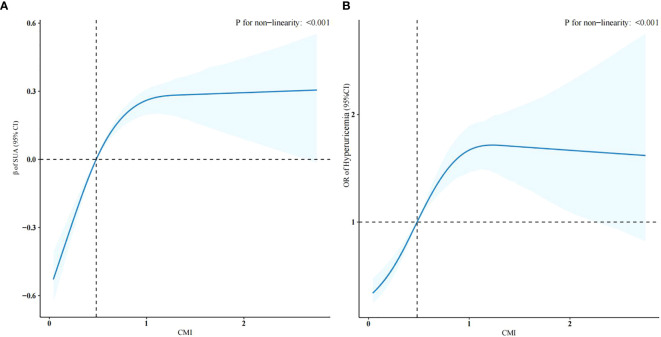
Restricted cubic spline regression curves of the relationships between the CMI and SUA levels **(A)** and hyperuricemia **(B)** (weighted).

The weighted linear regression analysis between the CMI and SUA levels showed a positive correlation (**Table 2**). The correlation remained after adjusting for all potential covariates (β = 0.28, 95% confidence interval (CI): 0.16–0.40), meaning that for each unit of the CMI, SUA increased by 0.28mg/dL. In Model 3, after adjusting for all covariates, the β coefficient for the upper two tertiles were 0.20 (CI: 0.09–0.31) and 0.51 (CI: 0.38–0.64) compared with the lowest tertile (*p* for trend, < 0.001). To further investigate the relationship between the CMI and SUA level, we created restricted cubic spline regression curves, which showed a nonlinear relationship, with participants with a higher CMI having higher SUA levels, and presumably the highest levels of uric acid at a CMI of around 1.5, as shown in **Figure 2A**.

**Table 2:** The relationship between CMI and the SUA level (weighted).

Variable	Model 1		Model 2		Model 3	
	β(95% CI)	*p* value	β(95% CI)	*p* value	β(95% CI)	*p* value
CMI Continuous	0.70 (0.56-0.85)	< 0.001	0.56 (0.42-0.69)	< 0.001	0.28 (0.16-0.40)	< 0.001
CMI Categories						
T1	0(Ref)		0(Ref)		0(Ref)	
T2	0.49 (0.37-0.62)	< 0.001	0.39 (0.29-0.49)	< 0.001	0.20 (0.09-0.31)	< 0.001
T3	1.13 (0.98-1.28)	< 0.001	0.93 (0.79-1.07)	< 0.001	0.51 (0.38-0.64)	< 0.001
*P* for trend		< 0.001		< 0.001		< 0.001

Model 1: Not adjusted.

Model 2: Adjusted by age, gender, race/ethnicity.

Model 3: Adjusted by age, gender, race/ethnicity, smoke, drink, BMI, TC, LDL-C, BUN, PIR, educationn level, marital status, CKD and CVD history.

**Table 3** shows the results of the logistic regression of the CMI and hyperuricemia. In model 3, which adjusted for all potential confounders, compared with the lowest CMI tertile, the highest CMI tertile had a more significant positive association with hyperuricemia (odds ratio (OR) =2.62, CI: 1.91–3.61, *p* < 0.001). This association was stably present in all three models (*p* for trend, < 0.001). **Figure 2B** shows that the risk of developing hyperuricemia was higher when the CMI was greater than 0.48.

**Table 3:** The relationship between CMI and hyperuricemia (weighted).

Variable	Model 1		Model 2		Model 3	
	OR(95% CI)	*p* value	OR(95% CI)	*p* value	OR(95% CI)	*p* value
CMI Continuous	2.33 (1.92-2.81)	< 0.001	2.30 (1.89-2.80)	< 0.001	1.54(1.26-1.88)	< 0.001
CMI Categories						
T1	1(Ref)		1(Ref)		1(Ref)	
T2	2.01 (1.55-2.61)	< 0.001	1.99 (1.52-2.62)	< 0.001	1.61 (1.17-2.21)	< 0.001
T3	4.60 (3.47-6.11)	< 0.001	4.60 (3.42-6.20)	< 0.001	2.62 (1.91-3.61)	< 0.001
*P* for trend		< 0.001		< 0.001		< 0.001

Model 1: Not adjusted.

Model 2: Adjusted by age, gender, race/ethnicity.

Model 3: Adjusted by age, gender, race/ethnicity, smoke, drink, BMI, TC, LDL-C, BUN, PIR, educationn level, marital status, CKD, and CVD history.

Additionally, a sensitivity analysis excluding participants using antihyperlipidemic agents revealed no significant impact on the association with SUA level and hyperuricemia outcomes, as detailed in **Supplementary Table S1**, **S2**.

### 3.3 Subgroup analyses

**Figure 3:**
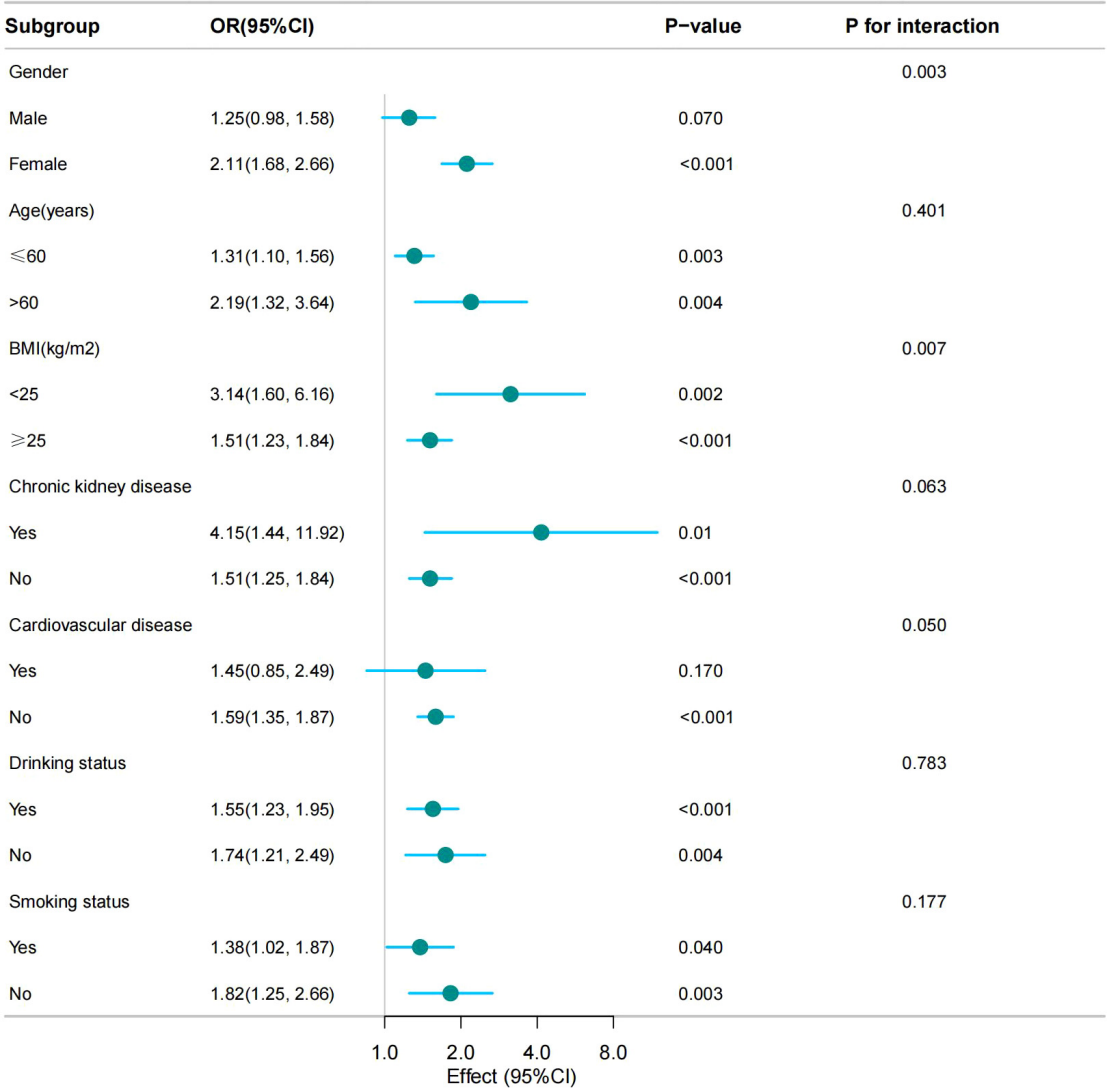
The association between the CMI and hyperuricemia by selected subgroups (weighted).

We conducted subgroup analyses based on stratification factors such as age, sex, BMI, smoking status, drinking status, CKD, and CVD to assess the stability of the relationship between the CMI and hyperuricemia. The results showed that the association of the CMI with hyperuricemia was stable present in all subgroups. Notably, no significant interactions were observed in the age, CKD, CVD, alcohol consumption, and smoking populations, indicating that the association did not depend on these variables (*p* for interaction, < 0.05). However, sex and BMI were found to significantly influence the strength of their association (*p* for interaction, < 0.05) (**Figure 3**).

## 4 Discussion

The present study examined the relationship between the CMI and SUA levels and hyperuricemia. After correcting for all confounders, the CMI was observed to correlate positively with SUA levels and hyperuricemia. This positive correlation was verified by smooth curve fitting. These results emphasize the relationship between central obesity and uric acid levels.

Subgroup analyses showed a higher incidence of hyperuricemia in the BMI <25 group than in the BMI ≥25 group, this observation may be attributed to two mechanisms: individuals with a BMI <25 may have reduced uric acid excretion due to low muscle mass, as shown in a US study revealed a negative correlation between uric acid levels and muscle mass (25). Besides, a subset of participants may exhibit “metabolically obese normal weight” phenotypes, characterized by excessive visceral adiposity despite normal BMI. This suggests that BMI is insufficient to fully predict the risk of hyperuricemia. We observed that the CMI in women exhibited a stronger correlation with hyperuricemia, which was similar to previous research reports (10, 26): First, estrogen can promote uric acid excretion and inhibit uric acid reabsorption. As a result, blood uric acid levels in premenopausal women are usually lower than in men of the same age. However, when women develop metabolic abnormalities (such as obesity, hyperlipidemia), the compensatory protective effect of estrogen may be broken, resulting in a sharp rise in uric acid levels and consequently amplifying the association between CMI and hyperuricemia in women. Besides, adult women have more adipose and subcutaneous fat tissue compared to men, with reported estimates at 39.9% body fat for adult women and 28% for men, respectively (27). Adipokines such as leptin and adiponectin secreted by adipose tissue can reduce uric acid excretion and enhance its production (28). In this study, participants using antihyperlipidemic agents were excluded due to their confounding effects on blood lipid profiles. The sensitivity analyze demonstrated the robustness of findings.

The correlation between the CMI and SUA was supported by the results of previous studies. Mao et al. (29) studied the relationship between obesity indices such as body roundness index, weight-adjusted waist index, and SUA levels using the NHANES database, reporting that central obesity is an independent risk factor for hyperuricemia. A positive correlation between SUA levels and serum LDL, total cholesterol, and triglyceride levels, and a negative correlation with HDL levels, was observed in Bangladeshi adults and American adolescents (30, 31). The incidence of hyperuricemia was shown to be higher in elderly Chinese people with high triglyceride levels and low HDL levels in a 6-year follow-up study (32). These studies demonstrated a strong link between blood uric acid levels and lipid metabolism. However, these indices failed to integrate body measurements with metabolic status; therefore, to better comprehend how central adiposity and hyperuricemia are related, we used the CMI as the independent variable. According to a study by Zhou et al. in China’s Yangtze River Delta region, the CMI, as a reliable index of visceral adiposity, and thus might be a useful monitoring indicator for the management of hyperuricemia in overweight people (11). Compared with other anthropometric measurements, the CMI had a stronger correlation with hyperuricemia in a cross-sectional investigation of asymptomatic persons with a normal BMI, as summarized by Liu et al. (16). While Zhou et al. focused on elderly cohorts, Liu et al. was conducted on patients with normal BMI. These findings cannot be generalized to the entire population because of to the limitations of the participants. Additionally, both of these studies were conducted in China. Our study highlighted the broader age coverage (20–80 years), included the full BMI spectrum. The present study confirmed that the CMI is positively associated with SUA levels and hyperuricemia in the US population. These studies have laid the foundation for preventing hyperuricemia by regulating the CMI.

The mechanism might be that excessive consumption of high-carbohydrate foods in obese individuals leads to overactive purine synthesis and uric acid production increases (33). Beyond this, increased visceral fat leads to an increase in free fatty acids in the portal system, hyperactivated hepatic fatty acid synthesis, and activation of the NADP NADPH mediated pathway of ribose 5-phosphate to phosphoribose pyrophosphate synthesis, leading to increased triglyceride synthesis and uric acid production (33). Furthermore, obesity affects the excretory function of the kidneys, resulting in decreased uric acid excretion. Obesity can lead to insulin resistance, which acts directly on the proximal tubular cells of the kidney, leading to sodium retention and acidification of urine, which in turn increases uric acid reabsorption and decreases excretion (34). Moreover, it has been suggested that keto acid produced during fat metabolism inhibits the excretion of SUA, thereby increasing SUA levels (35).

Therefore, in addition to assessing SUA level, we should also pay attention to the prevention and treatment of obesity and lipid metabolism disorders. Thus, health interventions, such as reasonable dietary structure, moderate exercise, and weight control, especially those focusing on the hip circumference, abdominal circumference growth changes, and lipid changes in patients with hyperuricemia, along with medication, might be able to reduce the incidence of hyperuricemia and improve the quality of survival of patients, even those with other cardiovascular, cerebrovascular, and metabolic diseases (36).

Our research is the first to assess the relationship between the CMI and hyperuricemia among adults in the USA. The sample size is large and thus our conclusions are convincing and realistic. Moreover, this research emphasizes that it is important for the general public and medical professionals to understand the impact of the CMI on SUA levels and hyperuricemia. The findings of this study extend the utility of CMI from a purely cardiovascular risk prediction tool to the screening of hyperuricemia. Its clinical applicability is reflected in the following aspects: First of all, CMI can be calculated using routine physical examination parameters (waist circumference, height, and lipid profiles), making it easy to incorporate into standard health check-ups without requiring additional specialized tests. This positions CMI as a practical and cost-effective indicator, suitable for widespread use. Then, In clinical practice, CMI serves as an effective tool for risk stratification, identifying individuals at potential risk even before their serum uric acid (SUA) levels reach diagnostic thresholds. Future guidelines may consider integrating CMI into the hyperuricemia risk assessment framework. Finally, CMI-guided treatment strategies may help lower SUA levels. This study suggests that targeting both components of CMI (fat reduction and lipid modulation) could simultaneously reduce SUA, offering a dual-intervention approach (e.g., lifestyle modifications addressing both obesity and dyslipidemia). These findings provide a theoretical foundation for designing future clinical trials using CMI as an intervention target for hyperuricemia treatment.

This study has several limitations. First, there are numerous possible factors influencing the CMI and hyperuricemia, and we were unable to adjust for all of them. In this study, we did not consider the effects of medication and diet; therefore, our findings should be validated by further research. Second, the study was a cross-sectional study, resulting in an inability to determine a causal association between CMI and hyperuricemia. Finally, the findings of this study might not be fully applicable to other countries because different nations and areas have distinct genetic backgrounds, habits, medical care standards, and socioeconomic circumstances. Future research should broaden the sample to include additional countries and areas to improve the generalizability and applicability of the findings.

## 5 Conclusion

Our results indicate a significant positive association between the CMI and hyperuricemia risk among US adults. This correlation was stable across multiple subgroups, and thus can be used to assess the potential risk of hyperuricemia prevalence in the general population.

## Data Availability

The datasets presented in this study can be found in online repositories. The names of the repository/repositories and accession number(s) can be found in the article/**Supplementary Material**.

## References

[1] Holmes EW Kelley WN Wyngaarden JB. The kidney and uric acid excretion in man. Kidney Int. (1972) 2:115–8. doi: 10.1038/ki.1972.81 4360739

[2] Li L Zhang Y Zeng C. Update on the epidemiology, genetics, and therapeutic options of hyperuricemia. Am J Transl Res. (2020) 12:3167–81. PMC7407685 32774692

[3] Joosten LAB Crişan TO Bjornstad P Johnson RJ. Asymptomatic hyperuricaemia: a silent activator of the innate immune system. Nat Rev Rheumatol. (2020) 16:75–86. doi: 10.1038/s41584-019-0334-3 31822862 PMC7075706

[4] Dalbeth N Gosling AL Gaffo A Abhishek A. Gout. Lancet. (2021) 397:1843–55. doi: 10.1016/S0140-6736(21)00569-9 33798500

[5] Johnson RJ Bakris GL Borghi C Chonchol MB Feldman D Lanaspa MA. Hyperuricemia, acute and chronic kidney disease, hypertension, and cardiovascular disease: report of a scientific workshop organized by the national kidney foundation. Am J Kidney Dis. (2018) 71:851–65. doi: 10.1053/j.ajkd.2017.12.009 PMC7286363 29496260

[6] Su H Liu T Li Y Fan Y Wang B Liu M. Serum uric acid and its change with the risk of type 2 diabetes: A prospective study in China. Prim Care Diabetes. (2021) 15:1002–6. doi: 10.1016/j.pcd.2021.06.010 34217642

[7] Copur S Demiray A Kanbay M. Uric acid in metabolic syndrome: Does uric acid have a definitive role? Eur J Internal Med. (2022) 103:4–12. doi: 10.1016/j.ejim.2022.04.022 35508444

[8] Berkowitz D. Blood lipid and uric acid interrelationships. JAMA. (1964) 190:856–8. doi: 10.1001/jama.1964.03070220062023 14202838

[9] Tana C Busetto L Di Vincenzo A Ricci F Ticinesi A Lauretani F. Management of hyperuricemia and gout in obese patients undergoing bariatric surgery. Postgrad Med. (2018) 130:523–35. doi: 10.1080/00325481.2018.1485444 29888674

[10] Su S-Y Lin T-H Liu Y-H Wu P-Y Huang J-C Su H-M. Sex difference in the associations among obesity-related indices with hyperuricemia in a large Taiwanese population study. Nutrients. (2023) 15:3419. doi: 10.3390/nu15153419 37571356 PMC10421218

[11] Liu XZ Li HH Huang S Zhao DB. Association between hyperuricemia and nontraditional adiposity indices. Clin Rheumatol. (2019) 38:1055–62. doi: 10.1007/s10067-018-4374-x 30498873

[12] Kim TH Lee SS Yoo JH Kim SR Yoo SJ Song HC. The relationship between the regional abdominal adipose tissue distribution and the serum uric acid levels in people with type 2 diabetes mellitus. Diabetol Metab Syndr. (2012) 4:3. doi: 10.1186/1758-5996-4-3 22301198 PMC3395847

[13] Wakabayashi I Daimon T. The “cardiometabolic index” as a new marker determined by adiposity and blood lipids for discrimination of diabetes mellitus. Clin Chim Acta. (2015) 438:274–8. doi: 10.1016/j.cca.2014.08.042 25199852

[14] Wu L Xu J. Relationship between cardiometabolic index and insulin resistance in patients with type 2 diabetes. DMSO. (2024) 17:305–15. doi: 10.2147/DMSO.S449374 PMC10821666 38283637

[15] Acosta García EJ Paez MC. Índice cardiometabólico como predictor de factores de riesgo cardiovascular en adolescentes. Rev salud pública. (2018) 20:340–5. doi: 10.15446/rsap.v20n3.61259 30844007

[16] Zuo Y-Q Gao Z-H Yin Y-L Yang X Feng P-Y. Association between the cardiometabolic index and hyperuricemia in an asymptomatic population with normal body mass index. IJGM. (2021) 14:8603–10. doi: 10.2147/IJGM.S340595 PMC8627282 34849005

[17] Wang H Sun Y Wang S Qian H Jia P Chen Y. Body adiposity index, lipid accumulation product, and cardiometabolic index reveal the contribution of adiposity phenotypes in the risk of hyperuricemia among Chinese rural population. Clin Rheumatol. (2018) 37:2221–31. doi: 10.1007/s10067-018-4143-x 29770928

[18] Liu Y Zhao W Liu X Jiang H Wu Y Luo L. Identifying reliable obesity indices for hyperuricemia among middle-aged and elderly populations: a longitudinal study. Lipids Health Dis. (2024) 26;23:305. doi: 10.1186/s12944-024-02296-6 PMC11426091 39327579

[19] Feig DI Kang D-H Johnson RJ. Uric acid and cardiovascular risk. N Engl J Med. (2008) 359:1811–21. doi: 10.1056/NEJMra0800885 PMC2684330 18946066

[20] Scinicariello F Przybyla J Carroll Y Eichwald J Decker J Breysse PN. Age and sex differences in hearing loss association with depressive symptoms: analyses of NHANES 2011–2012. Psychol Med. (2019) 49:962–8. doi: 10.1017/S0033291718001617 PMC8114788 29909806

[21] Liu X Gao W Yang J Mao G Lu H Xing W. Association between probiotic, prebiotic, and yogurt consumption and chronic kidney disease: The NHANES 2010–2020. Front Nutr. (2022) 9:1058238. doi: 10.3389/fnut.2022.1058238 36618701 PMC9822650

[22] Li C Meng T Wang B Liu C Jiang N Li J. Association between cardiometabolic index and asthma in adults: evidence from NHANES 2005–2018. J Asthma. (2024) 62(1):101–9. doi: 10.1080/02770903.2024.2388774 39105683

[23] Liu F You F Yang L Du X Li C Chen G. Nonlinear relationship between oxidative balance score and hyperuricemia: analyses of NHANES 2007–2018. Nutr J. (2024) 23:48. doi: 10.1186/s12937-024-00953-1 38704549 PMC11069158

[24] Song J Li Y Zhu J Liang J Xue S Zhu Z. Non-linear associations of cardiometabolic index with insulin resistance, impaired fasting glucose, and type 2 diabetes among US adults: a cross-sectional study. Front Endocrinol. (2024) 15:1341828. doi: 10.3389/fendo.2024.1341828 PMC10894973 38410697

[25] BeaversKMBeaversDPSerraMCBowdenRGWilsonRL. Low relative skeletal muscle mass indicative ofsarcopenia isassociated with elevations inserum uric acid levels: findings from NHANES III. J Nutr Health Aging. (2009) 13:177–82. doi: 10.1007/s12603-009-0054-5 19262948

[26] Gou R Dou D Tian M Chang X Zhao Y Meng X. Association between triglyceride glucose index and hyperuricemia: a new evidence from China and the United States. Front Endocrinol (Lausanne). (2024) 15:1403858. doi: 10.3389/fendo.2024.1403858 39010899 PMC11246899

[27] Perry AC Martin L. Race differences in obesity and its relationship to the sex hormone milieu. Horm Mol Biol Clin Invest. (2014) 19:151–61. doi: 10.1515/hmbci-2014-0004 25390023

[28] Wondmkun YT. Obesity, insulin resistance, and type 2 diabetes: associations and therapeutic implications. Diabetes Metab Syndr Obes. (2020) 13:3611–6. doi: 10.2147/DMSO.S275898 PMC7553667 33116712

[29] Mao T He Q Yang J Jia L Xu G. Relationship between gout, hyperuricemia, and obesity—does central obesity play a significant role?—a study based on the NHANES database. Diabetol Metab Syndr. (2024) 16:24. doi: 10.1186/s13098-024-01268-1 38254222 PMC10804703

[30] Ali N Rahman S Islam S Haque T Molla NH Sumon AH. The relationship between serum uric acid and lipid profile in Bangladeshi adults. BMC Cardiovasc Disord. (2019) 19:42. doi: 10.1186/s12872-019-1026-2 30791868 PMC6385393

[31] Shi Q Wang R Zhang H Shan Y Ye M Jia B. Association between serum uric acid and cardiovascular disease risk factors in adolescents in America: 2001-2018. PloS One. (2021) 16:e0254590. doi: 10.1371/journal.pone.0254590 34424900 PMC8382197

[32] Xu Y Dong H Zhang B Zhang J Ma Q Sun H. Association between dyslipidaemia and the risk of hyperuricaemia: a six-year longitudinal cohort study of elderly individuals in China. Ann Med. (2022) 54:2401–9. doi: 10.1080/07853890.2022.2118368 PMC9448407 36053052

[33] Ahmadieh H Azar S. Effects of sodium glucose cotransporter-2 inhibitors on serum uric acid in type 2 diabetes mellitus. Diabetes Technol Ther. (2017) 19:507–12. doi: 10.1089/dia.2017.0070 28749169

[34] Zong J Sun Y Zhang Y Yuan J Wang X Zhang R. Correlation between serum uric acid level and central body fat distribution in patients with type 2 diabetes. DMSO. (2020) 13:2521–31. doi: 10.2147/DMSO.S260891 PMC7372004 32765031

[35] Gil A. Uric acid is associated with features of insulin resistance syndrome in obese children at prepubertal stage. Nutricion Hospitalaria. (2009) 24(5):607–13. doi: 10.3305/nh.2009.24.5.4491 19893872

[36] Gaubert M Bardin T Cohen-Solal A Diévart F Fauvel J-P Guieu R. Hyperuricemia and hypertension, coronary artery disease, kidney disease: from concept to practice. IJMS. (2020) 21:4066. doi: 10.3390/ijms21114066 33561034 PMC7312288

