# Supplementary material for: The association between the cardiometabolic index and hyperuricemia: 2011–2016 NHANES
Source: Front Endocrinol (Lausanne). 2025 May 13;16:1545968. doi: 10.3389/fendo.2025.1545968 (PMC12106002; doi:10.3389/fendo.2025.1545968)
Supplement: Supplementary file 1 [file Table1.docx]

**Table S1.** The relationship between CMI and the SUA level excluding participants using antihyperlipidemic agents (weighted)

|  | Model 1 | | Model 2 | | Model 3 | |
| --- | --- | --- | --- | --- | --- | --- |
|  | β(95% CI) | *p* value | β(95% CI) | *p* value | β(95% CI) | *p* value |
| CMI Continuous | 0.78 (0.63-0.92) | < 0.001 | 0.56 (0.50-0.62) | < 0.001 | 0.33 (0.20-0.46) | < 0.001 |
| CMI Categories |  |  |  |  |  |  |
| T1 | 0(Ref) |  | 0(Ref) |  | 0(Ref) |  |
| T2 | 0.52 (0.40-0.64) | < 0.001 | 0.43 (0.34-0.52) | < 0.001 | 0.20 (0.08-0.32) | 0.002 |
| T3 | 1.18 (1.02-1.35) | < 0.001 | 0.94 (0.85-1.03) | < 0.001 | 0.54 (0.37-0.70) | < 0.001 |
| P for trend | 0 | < 0.001 |  | < 0.001 |  | < 0.001 |

Model 1: Not adjusted.

Model 2: Adjusted by age, gender, race/ethnicity.

Model 3: Adjusted by age, gender, race/ethnicity, smoke, drink, BMI, TC, LDL-C, BUN, PIR, educationn level, marital status, CKD and CVD history.

**Table S2.** The relationship between CMI and hyperuricemia excluding participants using antihyperlipidemic agents (weighted)

|  | Model 1 | | Model 2 | | Model 3 | |
| --- | --- | --- | --- | --- | --- | --- |
|  | OR(95% CI) | *p* value | OR(95% CI) | *p* value | OR(95% CI) | *p* value |
| CMI Continuous | 2.50 (2.13-2.92) | < 0.001 | 2.47 (2.09-2.90) | < 0.001 | 1.60 (1.32-1.95) | < 0.001 |
| CMI Categories |  |  |  |  |  |  |
| T1 | 1(Ref) |  | 1(Ref) |  | 1(Ref) |  |
| T2 | 1.86 (1.32-2.63) | < 0.001 | 1.84(1.29-2.63) | < 0.001 | 1.41 (0.94-2.13) | < 0.096 |
| T3 | 4.89 (3.54-6.75) | < 0.001 | 4.92 (3.50-6.92) | < 0.001 | 2.66 (1.80-3.93) | < 0.001 |
| P for trend |  | < 0.001 |  | < 0.001 |  | < 0.001 |

Model 1: Not adjusted.

Model 2: Adjusted by age, gender, race/ethnicity.

Model 3: Adjusted by age, gender, race/ethnicity, smoke, drink, BMI, TC, LDL-C, BUN, PIR, educationn level, marital status, CKD, and CVD history.
